# Effects of Electronic Cigarette Vaping on Cardiac and Vascular Function, and Post-myocardial Infarction Remodeling in Rats

**DOI:** 10.1007/s12012-024-09835-8

**Published:** 2024-02-10

**Authors:** Wangde Dai, Jianru Shi, Prabha Siddarth, Juan Carreno, Michael T. Kleinman, David A. Herman, Rebecca J. Arechavala, Samantha Renusch, Irene Hasen, Amanda Ting, Robert A. Kloner

**Affiliations:** 1 HMRI Cardiovascular Research Institute, Huntington Medical Research Institutes, 686 South Fair Oaks Avenue, Pasadena, CA 91105 USA; 2 Division of Cardiovascular Medicine of the Keck School of Medicine, University of Southern California, Los Angeles, CA 90017-2395 USA; 3 Department of Psychiatry, Jane and Terry Semel Institute for Neuroscience and Human Behavior, University of California, Los Angeles, CA 90095 USA; 4 Department of Environmental and Occupational Health, College of Health Sciences, University of California, Irvine, CA USA

**Keywords:** Electronic cigarettes, Myocardial infarction, Left ventricular remodeling

## Abstract

The effect of electronic cigarette (E-cig) vaping on cardiac and vascular function during the healing phase of myocardial infarction (MI), and post-MI remodeling was investigated. Sprague Dawley rats were subjected to left coronary artery ligation to induce MI. One week later, rats were randomized to receive either 12 weeks of exposure to purified air (*n* = 37) or E-cig vapor (15 mg/ml of nicotine) (*n* = 32). At 12 weeks, cardiac and vascular function, and post-MI remodeling were assessed. Baseline blood flow in the femoral artery did not differ between groups, but peak reperfusion blood flow was blunted in the E-cig group (1.59 ± 0.15 ml/min) vs. the air group (2.11 ± 0.18 ml/min; *p* = 0.034). Femoral artery diameter after reperfusion was narrower in the E-cig group (0.54 ± 0.02 mm) compared to the air group (0.60 ± 0.02 mm; *p* = 0.023). Postmortem left ventricular (LV) volumes were similar in the E-cig (0.69 ± 0.04 ml) and air groups (0.73 ± 0.04 ml; *p* = NS); and myocardial infarct expansion index did not differ between groups (1.4 ± 0.1 in E-cig group versus 1.3 ± 0.1 in air group; *p* = NS). LV fractional shortening by echo did not differ between groups at 12 weeks (E-cig at 29 ± 2% and air at 27 ± 1%; *p* = NS). Exposure to E-cig during the healing phase of MI was associated with altered vascular function with reduced femoral artery blood flow and diameter at reperfusion, but not with worsened LV dilation or worsened cardiac function.

**Supplementary Information:**

The online version contains supplementary material available at 10.1007/s12012-024-09835-8.

## Introduction

Electronic cigarettes (E-cig) are devices that can be used to deliver nicotine without combustion products, and their use has grown exponentially in the past decade. While probably being safer than smoking combustion cigarettes, many of the health implications with these devices remain unknown. Some of the specific components of E-cig vaping aerosols with known or suspected health effects, include nicotine, propylene glycol, and glycerol (and their heat degradation or other reaction products), various flavoring additives, sweeteners, and metals released from the heating elements (atomizer). Limited clinical studies have reported deleterious cardiovascular effects of E-cig vaping, including increases in blood pressure and heart rate, impaired endothelium-mediated dilation, vascular remodeling, and sympathetic overactivation. In addition, there have been reports of increased platelet activation, reactivity, and aggregation which have implications for heart attacks and strokes and strong associations of systemic inflammation and oxidative stress in vaping individuals (for review, see [1]). Animal studies have also provided experimental evidence that exposure to E-cig impairs cardiovascular function [2, 3].

Clinical studies reported that many patients with an acute coronary syndrome continue to smoke tobacco cigarettes after discharge from the hospital [4–6]. Continuous tobacco smoking after acute coronary syndromes was an independent predictor of total mortality and associated with greater mortality in patients [7, 8]. In recent years, the use of E-cig as a potential aid for smoking cessation has risen dramatically in post-acute coronary syndrome patients because they believe that E-cig are similarly or less harmful than FDA-approved tobacco smoking cessation treatments [9, 10]. Whether patients with myocardial infarction may be the same or less susceptible to the negative health effects of E-cig vaping as tobacco smoking remains unknown. Therefore, the purpose of the present study was to investigate the effect of E-cig exposure during the healing phase of myocardial infarction and to determine whether vaping during and after the immediate recovery period altered post-myocardial infarction remodeling and impaired cardiac and vascular function in an experimental left coronary artery permanent occlusion model in the rat. The clinical relevance of the permanent ligation of left coronary artery in rat was to mimic the patients in which timely reperfusion did not occur or reperfusion failed [11]. Although the reperfusion therapy is fundamental for ST-segment elevation myocardial infarction in clinical practice, the reperfusion rate varied among countries, for example, the reperfusion rate varied across geographical regions from 48.0% to 73.5% in China [12].

## Methods

All experimental protocols were approved by the Institutional Animal Care and Use Committees at the Huntington Medical Research Institutes, and the University of California, Irvine. Both institutes are accredited by the Association for Assessment and Accreditation of Laboratory Animal Care International. The experiments were performed in accordance with the “Guide for the Care and Use of Laboratory Animals” (NIH publication No. 85–23, National Academy Press, Washington DC, revised 2011).

Myocardial infarction (MI) was performed by ligation of the proximal left coronary artery through a left thoracotomy under anesthesia with ketamine (90 mg/kg i.p.) and xylazine (10 mg/kg i.p.) in Sprague Dawley rats (10 weeks old, both sexes) as described previously [13]. In brief, a left thoracotomy was performed to expose the heart. The pericardium was opened, and the proximal left coronary artery was encircled with a 4–0 silk suture and permanently ligated. Successful occlusion was confirmed by cyanosis and akinesis of the LV anterior wall. The left thoracotomy was then closed in layers, and the rat was allowed to recover from anesthesia and returned to a clean cage.

At one week after MI, rats were randomized to receive either 12 weeks of exposure to purified air (*n* = 20 male/17 female) or E-cig vapor plus 15 mg/ml of nicotine (*n* = 16 male/16 female) using the methods described previously [14]. In brief, at the Air Pollution Health Effects Laboratory at the University of California, Irvine, the rats were held in individual exposure tubes using a nose-only inhalation exposure system (In-Tox Products, Clinton MS) to minimize dermal exposure. The exposure tubes were connected to an exposure manifold, into which a custom-made vaping system injected E-cig aerosols using a puff protocol mimicking a standard human protocol with a puff frequency of 2 s per 30 s (2 puffs/minute) with a dilution flow rate of 1.67 L per minute (equating to a 35 mL puff volume as described in the International Organization for Standardization (ISO) smoking conditions). The aerosol was delivered by individual jets to the nose of each animal. A series of exhaust ports in the front of each animal nose cone eliminated exhaled air and minimized the potential re-breathing with CO_2_ buildup. The rats were exposed to air or E-cig aerosol 5 h/day, 4 days/week (Monday through Thursday) for a total of 12 weeks.

The E-cig vaporizer contained a 50/50 (vol/vol) propylene glycol/vegetable glycerin (PG/VG) matrix to which were added tobacco flavor (www.VaporFi.Com) and 15 mg/mL pure nicotine (L-Nicotine, Acros Organics, Lot: A0382410). The vaporizers used were commercially available mod-style vape units with stainless steel atomizers, and the 15 mg/mL dosage of nicotine matched for the original NIDA Standard Research E-cigarette (SREC) used for many human exposures.

The rats were exposed for 12 weeks, after which they were transferred to Huntington Medical Research Institutes (Pasadena, CA) and allowed to acclimate for 2 to 4 days before cardiovascular function assessment. The rats were anesthetized with an intraperitoneal injection of ketamine (90 mg/kg) and xylazine (10 mg/kg), intubated and then mechanically ventilated. The chest and neck areas were shaved, and an echocardiogram was obtained (15-MHz transducer and Sonos 5500 ultrasound system, Philips Medical System, Andover, MA). LV end-diastolic and end-systolic internal diameters were measured at the mid-papillary muscle level, and LV fractional shortening was calculated. A 2F high-fidelity catheter-tipped micromanometer (model SPR-869, Millar, Inc.) was inserted into the right carotid artery and advanced into the ascending aorta to obtain the arterial blood pressure (BP) and heart rate (HR). The catheter-tipped micromanometer was then advanced into the left ventricular (LV) cavity to measure LV end-systolic pressure (LVESP), LV end-diastolic pressure (LVEDP), the maximal slope of LV systolic pressure increment (dP/dtmax), diastolic pressure decrement (dP/dtmin), and Tau (a measure of diastolic function).

Cardiac output was measured using a thermodilution technique previously described [15]. In brief, a thermocouple catheter was introduced into the right carotid artery and advanced to the aortic arch, and the tip of the temperature probe remained positioned in the aortic arch immediately distal to the aortic valve. A catheter (PE-50) was inserted through the right jugular vein and its tip placed into the right atrium for future bolus saline injection. Cold saline solution (0 °C, 0.2 ml) was injected into the right atrium through the catheter within the jugular vein and the thermodilution curve was recorded. Cardiac output was measured using the PowerLab system (LabChart 8.0, PowerLab, ADInstruments). The results of 3 measurements were averaged for each animal.

After completion of the aforementioned cardiac function evaluation and still under anesthesia, the vascular function was assessed through measurement of internal diameter, blood flow velocity, and flow rate in the left femoral artery. The femoral artery was occluded by placing the blood pressure cuff at the inguinal level, and ischemia was induced by inflating the cuff to 200 mmHg for 5 min according to the methods introduced by Brandli [16]. The cuff was rapidly deflated to induce a brief high-flow reperfusion state through the femoral artery (reactive hyperemia). The baseline femoral artery diameter and blood flow velocity were measured prior to occlusion and again at 3 min after artery reperfusion. The femoral artery diameter was measured using a M-mode display, and the blood flow velocity was measured using Doppler signal with high-resolution ultrasound (15-MHz transducer and Sonos 5500 ultrasound system, Philips Medical System, Andover, MA). The femoral artery blood flow rate (mL/min) was measured using an ultrasound perivascular flow probe (TS410, Transonic Systems Inc., NY, USA) using methods described by Kenwright et al. [17]. In brief, a transverse incision (~ 2 cm) was made below the left inguinal ligament, and a short segment of the left femoral artery distal to the superficial circumflex iliac artery was carefully exposed and isolated from the nerve and vein using fine forceps. Ultrasound gel was applied to the ultrasonic flow probe's sensor, which was then attached to the exposed segment of the femoral artery.  Femoral artery flow rate measurements were continuously recorded during resting conditions, during a 5 minutes acute clamping of the left femoral artery, and during 5 minutes after reperfusion.

Following the cardiovascular assessments, 1 mL of potassium chloride (149 mg/mL) was intravenously injected to stop the hearts in their relatively diastolic or relaxed state while the rats were under deep anesthesia. The rats were weighed. The tibia length was measured. The hearts were excised, washed in saline, and weighed. The left and right ventricles were weighed separately. The LVs were pressure fixed (pressure equal to 13 cm water column) in formalin, and the volumes of LV were measured by filling the cavity with water and weighing, repeated three times. The lungs were harvested, and the lung wet/dry weight was determined.

Four hearts in each group were flush frozen for future biochemistry studies; all other hearts were fixed for histology. Formalin-fixed hearts were cut into 3 transverse slices. The middle slice was embedded in paraffin for histology. The paraffin embedded tissue was sectioned (5 μm thickness) and stained with hematoxylin and eosin, and picrosirius red. Histological images of the stained sections were traced with computerized planimetry and the following parameters were measured: (1) scar thickness (average of 5 equidistant measurements) and septum thickness (average of 3 equidistant measurements); (2) Total LV epicardial circumference and endocardial circumference; and (3) circumference occupied by infarcted wall (infarct size was expressed as percentage of total LV circumference); (4) expansion index, as defined by Hochman and Choo [5], which is expressed as [LV cavity area/total LV area × septum thickness/scar thickness].

### Statistical Analysis

Prior to analyses, data were first inspected for normality, homogeneity of variance, and other assumptions to ensure suitability for parametric statistical tests. All data were reported as mean ± SEM. Outcome measures were compared between the 2 exposure groups using Student's t-test. The significance threshold was set at *p* < 0.05. We also present effect size estimates (Cohen’s d) for all comparisons. In order to examine whether there were sex-dependent effects of vaping, we conducted ANOVAs for all outcomes, where we included exposure group, sex, and the interaction between exposure group and sex as predictors. Since these analyses were not a priori hypothesized, we consider these as exploratory; hence, we present the results of these analyses (data in 4 groups: (1) air/male; (2) air/female; (3) E-cig + /male; and (4) E-cig + /female) in Supplementary Material.

## Results

A total of 40 male and 40 female rats were used in this study. Four out of 40 male (10%) and 7 out of 40 (17.5%) female rats died within 1 day after left coronary artery ligation. Therefore, 36 male and 33 female rats were randomized to receive either exposure to purified air or E-cig vapor. There were no rats died during the air or E-cig exposure period.

Cardiac function was assessed by echocardiogram 12 weeks after the E-cig exposures. The data (Table 1) demonstrate that LV end-diastolic internal diameter, LV end-systolic internal diameter, LV fractional shortening, and left ventricular wall thickness were all comparable between the 2 groups. Cardiac output measured by thermodilution was similar in E-cig group (44.6 ± 2.5 ml/min) and control group (42.1 ± 1.5 ml/min, *p* = 0.4).

**Table 1:** Cardiac function assessed by echocardiography at 12 weeks of air or E-cig exposure

	Air	E-cig Nic +	*p* value	Effect size
	*n* = 37	*n* = 32		(Cohen’s d)
LV Diastolic ID (mm)	7.85 ± 0.18	8.03 ± 0.17	0.478	0.17
LV Systolic ID (mm)	5.74 ± 0.17	5.77 ± 0.23	0.918	0.03
LVFS	27.11 ± 1.00	28.85 ± 1.75	0.395	0.21
LV systolic wall thickness (mm)	3.74 ± 0.14	3.88 ± 0.18	0.562	0.14
LV diastolic wall thickness (mm)	2.91 ± 0.12	2.87 ± 0.14	0.835	0.05

*LV* left ventricular, *ID* internal diameter, *LVFS* left ventricular fractional shortening

Hemodynamic data (Table 2) acquired by catheter-tipped micromanometer showed that arterial blood pressure was significantly reduced after E-cig exposure. There were no significant differences in heart rate, LV end-diastolic pressure (Ped), LV end-systolic pressure (Pes), dP/dt max, dP/dt min, or Tau between the exposed and control groups.

**Table 2:** Hemodynamic parameters at 12 weeks of air or E-cig exposure

	Air	E-cig Nic +	*p* value	Effect size
	*n* = 37	*n* = 32		(Cohen’s d)
Systolic Pressure (mmHg)	87 ± 2	81 ± 2	0.023	0.55
Diastolic Pressure (mmHg)	68 ± 2	62 ± 2	0.004	0.73
Mean Pressure (mmHg)	77 ± 2	70 ± 2	0.010	0.63
Pulse Pressure (mmHg)	18 ± 1	19 ± 1	0.425	− 0.20
Heart Rate (BPM)	233 ± 6	220 ± 7	0.157	0.35
LV Pes (mmHg)	85 ± 2	87 ± 3	0.659	− 0.11
LV Ped (mmHg)	5 ± 0	5 ± 0	0.867	0.04
dP/dt max (mmHg/s)	4446 ± 132	4692 ± 144	0.230	− 0.29
dP/dt min (mmHg/s)	3674 ± 113	3808 ± 172	0.537	− 0.15
Tau (ms)	15.6 ± 0.5	15.8 ± 0.6	0.775	− 0.07

*BPM* beats per minute, *LV Pes* left ventricular end-systolic pressure, *LV Ped* left ventricular end-diastolic pressure

**Fig. 1:**
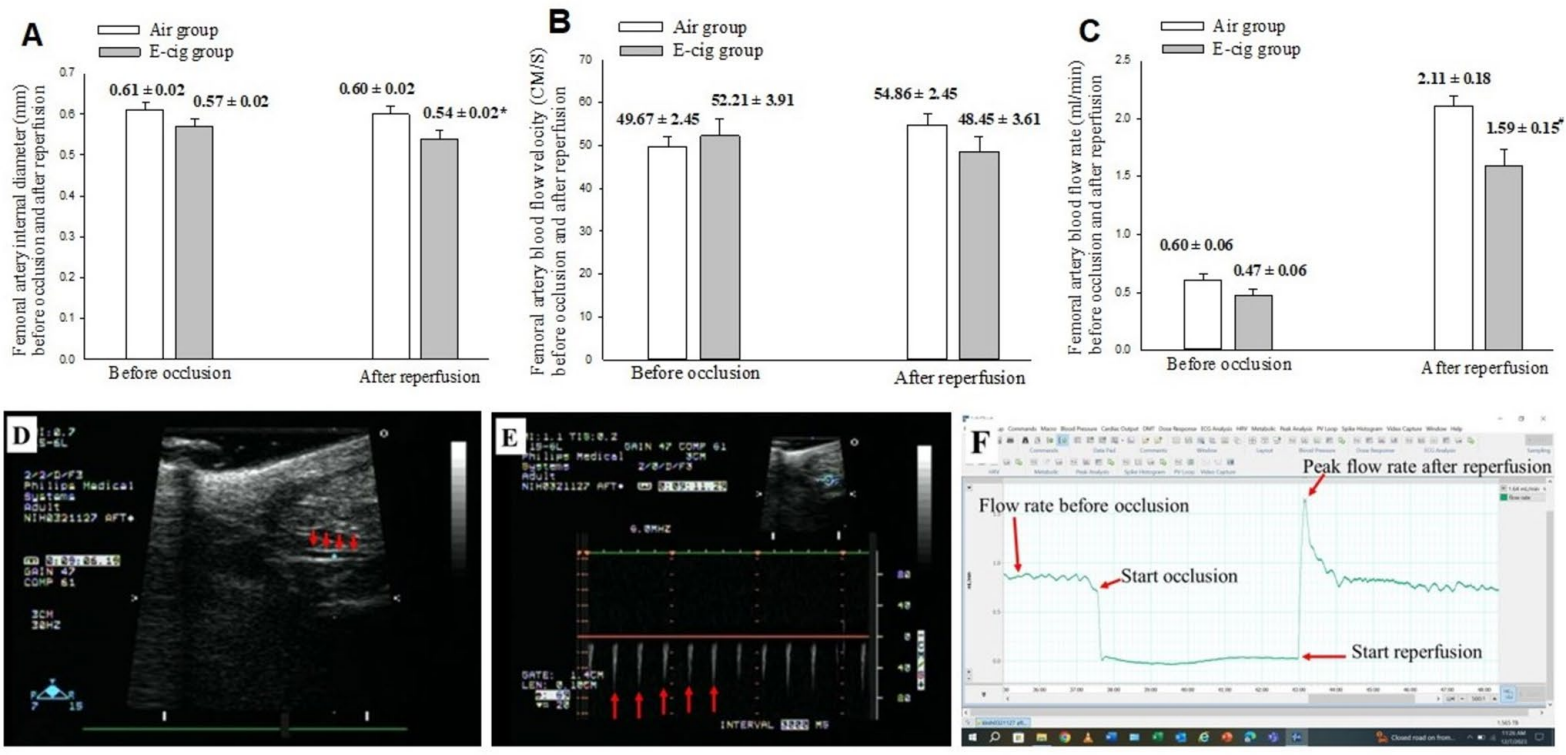
The femoral artery diameter (mm) was measured using a M-mode display (panel D), and blood flow velocity (CM/S) was measured using Doppler signal (panel E) with high-resolution ultrasound (15-MHz transducer and Sonos 5500 ultrasound system, Philips Medical System, Andover, MA). The femoral artery blood flow rate (mL/min) was measured using an ultrasound perivascular flow probe (panel F) (TS410, Transonic Systems Inc., NY, USA). **A** The femoral artery internal diameter (mm) before artery occlusion and at 3 min after reperfusion. There was a significantly smaller diameter (*: *p* = 0.023) in the E-cig compared with air exposure group. **B** The femoral artery blood flow velocity (CM/s) before artery occlusion and at 3 min after reperfusion. **C** The femoral artery blood flow rate (ml/min) before artery occlusion and after reperfusion (peak flow rate). The peak blood flow rate was measured immediately after reperfusion. There was a significantly lower flow rate (#: *p* = 0.034) in the E-cig compared with air exposure group. **D** Representative picture of femoral artery (red arrows). **E** Doppler signal of flow velocity of femoral artery (red arrows). **F** Continuous tracing recording of flow rate at baseline before femoral artery occlusion, during 5 min of occlusion, and after reperfusion (red arrows point different time points, separately)

Vascular function was assessed by measurements of internal diameter, blood flow velocity, and flow rate in the left femoral artery in femoral artery, and the data are presented in Table 3 and Fig. 1. Femoral artery diameter measured by echo M-mode was comparable in the E-cig group (0.57 ± 0.02 mm) and air group (0.61 ± 0.02 mm) before artery occlusion but was narrower in the E-cig group (0.54 ± 0.02 mm) compared to the air group (0.60 ± 0.02 mm; *p* = 0.023) after reperfusion. Baseline blood flow rate measured by ultrasound perivascular flow probe technology did not differ between E-cig group (0.47 ± 0.06 ml/min) and air group (0.60 ± 0.06 ml/min, *p* = 0.134); but, post-occlusion, the peak reperfusion blood flow rate was blunted in the E-cig group (1.59 ± 0.15 ml/min) vs. the air group (2.11 ± 0.18 ml/min; *p* = 0.034).

**Table 3:** Parameters of vascular function assessed by flow-mediated vasodilation of femoral artery at 12 weeks of air or E-cig exposure

	Air	E-cig Nic +	*p* value	Effect size
	*n* = 37	*n* = 32		(Cohen’s d)
Artery ID (mm) before occlusion	0.61 ± 0.02	0.57 ± 0.02	0.213	0.31
Flow velocity (CM/S) before occlusion	49.67 ± 2.45	52.21 ± 3.91	0.585	− 0.14
Flow rate (ml/min) before occlusion	0.60 ± 0.06	0.47 ± 0.06	0.134	0.36
Artery ID (mm) after reperfusion	0.60 ± 0.02	0.54 ± 0.02	0.023	0.56
Flow velocity (CM/S) after reperfusion	54.86 ± 3.29	48.45 ± 3.61	0.197	0.32
Peak flow rate (ml/min) after reperfusion	2.11 ± 0.18	1.59 ± 0.15	0.034	0.51
Δ% of artery ID	39 ± 2%	59 ± 15%	0.020	0.58
Δ% of flow velocity	46 ± 2%	47 ± 4%	0.205	0.31
Δ% of flow rate	111 ± 18%	59 ± 15%	0.034	0.51

Δ% = percentage of change from baseline to post-reperfusion

Postmortem parameters including LV volume, body weight, heart weight, tibia length, lung wet, and dry weight are summarized in Table 4. The E-cig group tended to have lower values than the control group; however, other than the right ventricular weight, the differences between the 2 groups were not statistically significant.

**Table 4:** Postmortem parameters at 12 weeks of air or E-cig exposure

	Air	E-cig Nic +	*p* value	Effect size
	*n* = 33	*n* = 28		(Cohen’s d)
Postmortem LV volume (ml)	0.7333 ± 0.0373	0.6874 ± 0.0404	0.407	0.21
BW (grams)	415 ± 22	368 ± 18	0.109	0.41
Tibia length(mm)	43.0576 ± 0.5202	41.7075 ± 0.5151	0.070	0.47
Heart weight (grams)	1.1277 ± 0.0541	1.0118 ± 0.0463	0.109	0.41
Left ventricle weight (grams)	0.9372 ± 0.0450	0.8488 ± 0.0387	0.142	0.38
Right Ventricle Weight (grams)	0.1923 ± 0.0106	0.1614 ± 0.0095	0.034	0.55
Heart weight/BW	0.0028 ± 0.0001	0.0028 ± 0.0001	0.779	0.07
LV weight/BW	0.0023 ± 0.0001	0.0023 ± 0.0001	0.550	0.16
Heart weight/Tibia	0.0259 ± 0.0010	0.0241 ± 0.0009	0.181	0.34
LW weight/tibia	0.0215 ± 0.0009	0.0202 ± 0.0008	0.252	0.29
Lung wet weight (grams)	1.3556 ± 0.0438	1.3724 ± 0.0530	0.808	0.06
Lung dry weight (grams)	0.2110 ± 0.0080	0.2052 ± 0.0069	0.586	0.14
Lung wet/dry weight ratio	6.4863 ± 0.1059	6.7351 ± 0.2047	0.287	− 0.29

*LV* left ventricular, *BW* Body weight

**Fig. 2:**
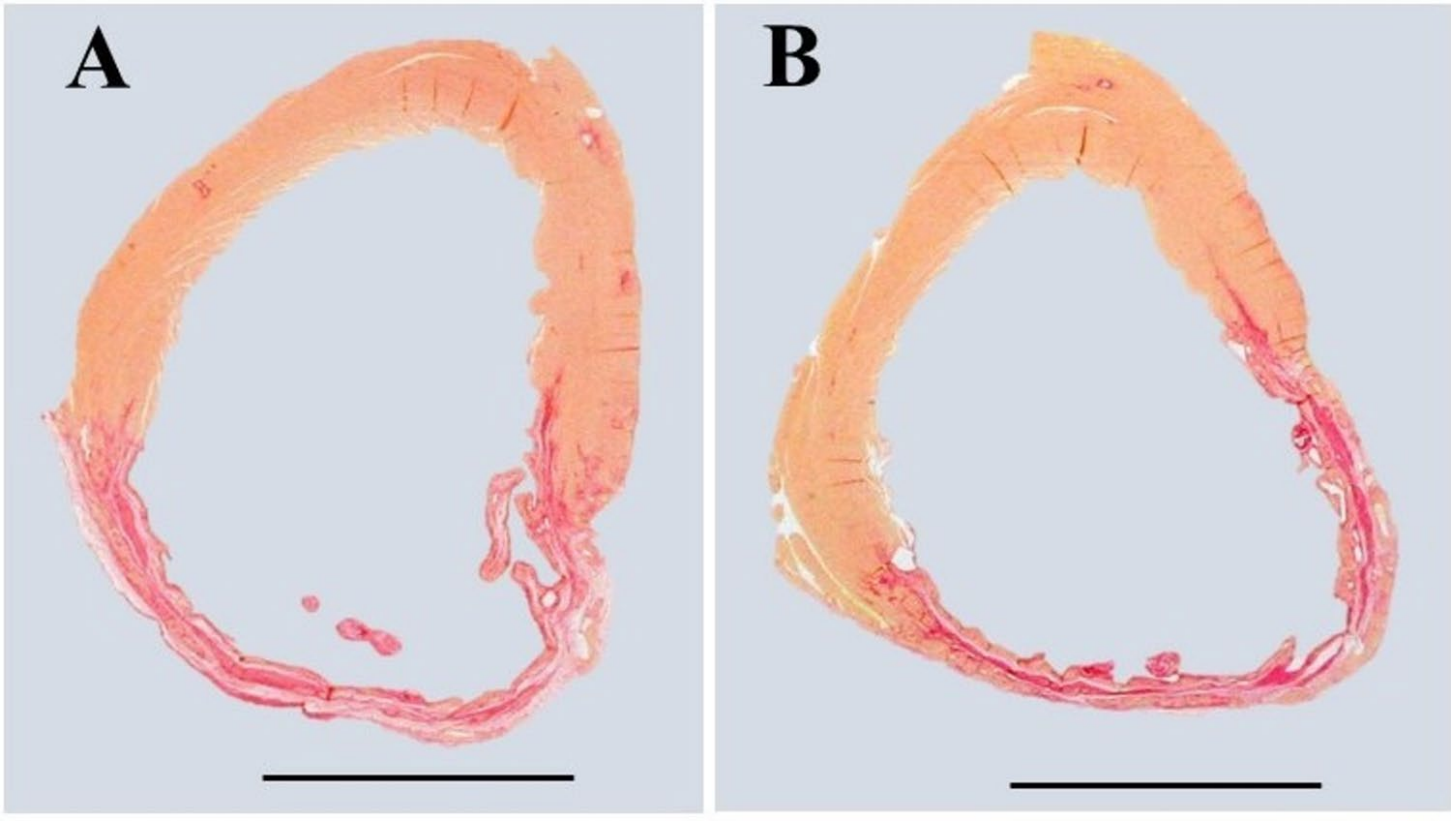
Representative slices of hearts stained with picrosirius red staining. Muscle cells stain yellow while collagen (scar) stains red. **A** Air-exposed heart with myocardial infarct scar as control; **B** E-cig-exposed heart with myocardial infarct scar. Note that scar circumference was comparable in the air-exposed heart compared to the E-cig-exposed heart, and the scar thickness and total LV circumference were also comparable between the two groups. (Scale bar = 5 mm)

**Fig. 3:**
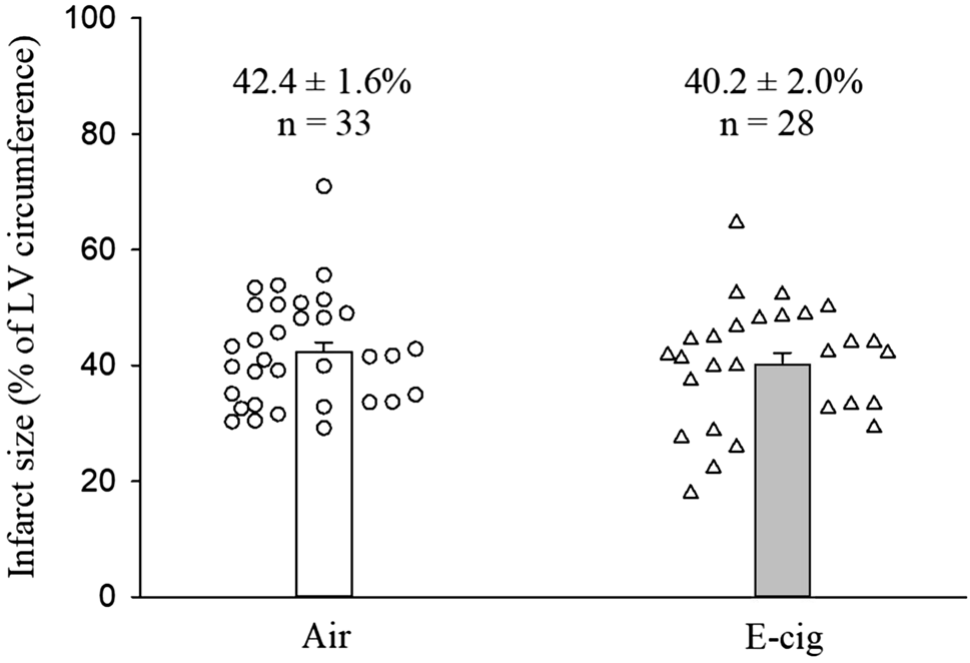
Infarct size, expressed as % of left ventricular circumference, was comparable between air or E-cig group after 12 weeks of exposure. The infarct size varied from 29.2% to 71.0% in the air group, and from 17.9% to 64.7% in the E-cig group. The inherent variability of infarction size of in rat model is caused by the facts of the incapacity of occluding the left coronary artery exactly at the same point in all rats, as well as the anatomic variations of coronary artery among the rats

Table 5 summarizes the histological data of the post-infarcted heart (Fig. 2). The results showed that infarct size (% of LV circumference, Fig. 3) and infarct expansion index, as well as the LV infarcted and non-infarcted wall thickness were similar between the 2 groups.

**Table 5:** Post-infarction LV remodeling parameters at 12 weeks of air or E-cig exposure

	Air	E-cig Nic +	*p* value	Effect size
	*n* = 33	*n* = 28		(Cohen’s d)
Total LV area (mm^2^)	98.5 ± 3.7	93.6 ± 3.1	0.310	0.26
LV cavity area (mm^2^)	48.7 ± 2.6	44.7 ± 2.1	0.239	0.30
Infarct size (% of LV circumference)	42.4 ± 1.6	40.2 ± 2.0	0.391	0.22
LV infarcted wall thickness (mm)	0.78 ± 0.03	0.78 ± 0.06	0.998	0.00
LV non-infarcted wall thickness (mm)	1.98 ± 0.05	2.02 ± 0.06	0.558	− 0.15
Infarct expansion index	1.3 ± 0.1	1.4 ± 0.1	0.481	− 0.18

*LV* left ventricular

Exploratory analyses examining interaction of sex and exposure group on effects of vaping are presented in the Supplementary Material. Most measures were not differentially affected in males vs. females.

## Discussion

The main findings of the present study are that exposure to E-cig with nicotine during healing phase of MI did not worsen post-MI remodeling and cardiac function but altered vascular function with reduced femoral artery blood flow and diameter at reperfusion.

E-cig vaping is known to cause vascular dysfunction in healthy humans and animals [18]. Mohammadi et al. [19] recruited a total of 120 healthy volunteers to assess the effects of chronic E-cig use (current use of E-cig > 5 times/week for > 3 months) on vascular function. Arterial flow-mediated dilation was calculated in 51 participants using a standard clinical ultrasound-based method. After the baseline B-mode ultrasound images of the brachial artery and spectral Doppler images of flow velocity were recorded, a forearm cuff was inflated to 250 mmHg for five minutes to induce transient ischemia. Then the cuff was deflated, and Doppler images were immediately obtained to measure reactive hyperemia. The results demonstrated that vascular function was injured in E-cig users relative to the nonusers. Olfert et al. [2] randomly subjected healthy female mice (C57BL/6) to chronic daily exposure to E-cig vapor, standard cigarette smoke, or filtered air for 8 months. Changes in thoracic aortic tension in response to vasoactive-inducing compounds were measured using ex vivo wire tension myography and force transduction after 8-mo exposure. Chronic exposure to E-cig vapor significantly impairs aortic endothelial function and induces cardiovascular dysfunction. Consistent with these studies, our present results also support that chronic E-cig exposure impaired the vascular function in the post-MI healing phase in rats. Note that in our experimental myocardial infarction model, post-MI pathophysiological conditions itself can induce vascular dysfunction [20]. The underlying mechanism may involve the decrease in the bioavailability of NO, accumulation of ROS, chronic low-grade inflammation, and enhanced sympathetic vasomotor tone, and needs to be further investigated.

Martinez-Morata et al. [21] summarized 14 published E-cig studies (including 13 experimental studies and 1 observational study) that evaluated the potential effects of E-cig use on systolic and diastolic blood pressure. In all experimental studies, systolic and diastolic blood pressure levels were measured at pre-intervention baseline and up to 4 h after the intervention. The results demonstrated that the short-term effects of E-cig use showed a consistent increase of blood pressure immediately to several hours after exposure to e-cigs containing nicotine. The short-term increases in blood pressure for the nicotine E-cig groups may be due to the activation of the sympathetic nervous system mediated by nicotine in the e-cig aerosol that is dependent on plasma levels of nicotine. The sympathetic activation is dependent on concentration of plasma nicotine. In our present study, arterial blood pressure went down in the E-cig group. The explanation may be that blood pressure was measured at 2–4 days after the last vaping. Our results were consistent with the observed reduction in blood pressure after the first few days of smoking cessation [22]. Ward et al. [22] reported that both systolic blood pressure (SBP) and diastolic blood pressure (DBP) decreased from baseline to day 8 of post-smoking cessation and then increased back toward baseline from days 8 to 90 days. This suggested that the initial reduction in blood pressure after cessation may be due to the cardiovascular system making functional adaptations to chronic nicotine intake and requiring a period of time after cessation to achieve a new homeostasis. Another explanation is that the acute response to E-cig exposure on blood pressure may be very different than a chronic response. After an acute first exposure of E-cig, the body may respond with a sympathetic response with an elevation of BP. However, after chronic exposure, the body may become desensitized and there is less of a sympathetic response.

Experimental studies provide evidence that tobacco smoking is harmful to post-MI remodeling and recommended smoking cessation. Duarte et al. [23] induced MI in Wistar rats by ligation of the left coronary artery. Two days after MI, the rats were exposed to tobacco smoke for 6 months. After 6 months of exposure, echocardiographic and histological data demonstrated that tobacco smoke significantly intensified left ventricular remodeling following MI compared with controls. In contrast, our current study demonstrated that exposure to E-cig with nicotine during healing phase of MI did not worsen post-MI remodeling and cardiac function in rats. Our data may seem to provide evidence for the use of E-cigs among post-acute coronary syndrome patients. These patients perceived E-cigs as less harmful to cardiac health than tobacco smoking [24]. However, in the literature, there are no existing data regarding the safety of E-cig use among the post-acute coronary syndrome patients. We  cannot conclude from our present data that E-cig could be used as tobacco-cessation products for the post-acute coronary syndrome patients, because (1) our present data showed that E-cig exposure negatively affected endothelial function, suggesting vascular dysfunction [24]; (2) our 12 weeks of E-cig exposure is relatively short, long-term studies, such as 1 year of exposure or longer as the full length of a rat lifetime, are needed to delineate whether E-cig use is less hazardous to cardiovascular health than cigarette smoking [25]; (3) the components of electronic nicotine delivery system products contain various kind of flavors, have different types of device and device features (e.g., power, voltage), and add different concentration of nicotine. These variables may be expected to affect the impacts of E-cig on cardiovascular health and should be considered in future experimental design [1]; (4) young healthy rats were used in the present study. It is important to note that post-acute coronary syndrome patients are often older and with multiple co-morbidities, such as diabetes mellitus, hypertension, atherosclerosis, etc; (5): there are no existing clinical studies that have assessed the impact of E-cig exposure on post-acute coronary syndrome patients. Large clinical survey studies are needed. The safety of nicotine-containing E-cig as tobacco-cessation products in post-acute coronary syndrome patients remains to be determined and needs more investigations in both clinical and experimental situation.

Our research group [14] previously reported the effects of chronic E-cig usage on acute myocardial infarct size. In that study, rats were exposed to E-cig vaping for 8 weeks, followed by 30 min of left coronary artery occlusion and 3 h of reperfusion. This study mimicked an E-cig user who then develops an acute myocardial infarction. The results demonstrated that 8-week exposure to E-cig vaping with or without nicotine did not increase myocardial infarct size or worsen the no-reflow phenomenon in the setting of acute myocardial infarction. However, the relatively short-term E-cig exposure did induce cardiovascular dysfunction including reduced cardiac output and reduced LV positive and negative dp/dt, consistent with a reduction in cardiac contractility and relaxation, and increased systemic arterial resistance in these rats after coronary artery occlusion and reperfusion. In contrast, E-cig exposure starting at 1 week after myocardial infarction did not affect cardiac function assessed by echocardiography, thermodilution technique, and catheter-tipped micromanometer in our present study. The differences between the present results and our previous results can be explained by the fact that healthy rats were exposed to E-cig in our previous study, while rats with myocardial infarction were exposed to E-cig in the present study. Myocardial infarction injury itself can severely affect the cardiac and vascular function.

Whether cigarette smoking or vaping affects the post-MI remodeling in a sex-dependent way remains controversial. Kaplan et al. [3] exposed male and female C57BL/6 J mice (5 months old) to 3R4F cigarettes smoking for 2 weeks, followed by surgical permanent left coronary artery ligation and then 1 additional week post-MI smoking. In this study, compared to their nonsmoking counterpart, cigarette smoking significantly worsened both left and right ventricular remodeling only in males, but not in females, within 7 days of post-MI. Our exploratory analyses examining sex differences indicated that vaping did not affect the post-MI remodeling in a sex-dependent way. The difference between our current findings and Kaplan’s study may be related to different species (mice and rat) exposed to different smoking (cigarette and E-cig) in different exposure protocols. We emphasize that these findings are preliminary and need to be validated in a future study designed to examine sex differences upon cigarette and E-cig exposure.

There are some limitations in our study. First, we only measured arterial blood pressure, cardiac and vascular functions one time at the end of study, and did not follow the cardiovascular function changes before and after MI (before and after E-cig exposure). Second, we used a permanent ligation of the left coronary artery model, not a more clinically relevant ischemia/reperfusion model. However, the key goal of our present study design aimed to determine whether E-cig exposure could worsen the post-myocardial infarction remodeling, cardiac and vascular function. The rats were subjected to permanent ligation of left coronary artery to induce myocardial infarction model. This is a model known to induce remodeling of the left ventricle and that is why we chose it. It results in consistent substantial infarct with a well-delineated scar and expansion of the infarct, and our group has had extensive experience with this model and it is very reproducible [26, 27]. On the other hand, in clinical practices, reperfusion therapy is performed in only ~ 70% of patients after heart attack. There are ~ 30% of patients in which timely reperfusion did not occur or reperfusion failed. Therefore, surgical permanent occlusion of the left descending coronary artery in rats, modeling acute STEMI in patients, is also commonly widely used to model these ~ 30% of patients (For review, please read [11]). Third, in this particular study we did not include a group of healthy rats exposed to e-cigarettes; however, in previous studies we did examine the effect of e-cigarettes on otherwise healthy rats.

## Conclusions

Nicotine-containing E-cig is often touted as a potential tobacco-cessation product. Our present study provides evidence that exposure to E-cig during the healing phase of MI was associated with altered vascular function with reduced femoral artery blood flow and diameter at reperfusion, but not with worsened LV dilation or worsened cardiac function. Although our present results and other studies [25, 28] suggest that E-cig may be less hazardous to cardiovascular health than tobacco smoking, and some reports favor E-cig helping smokers quit tobacco [9], the utility of E-cig for long-term tobacco smoking cessation remains to be determined and needs further experimental and clinical studies.

### Supplementary Information

Below is the link to the electronic supplementary material.

Supplementary file1 (DOCX 27 KB)

## Data Availability

The data collected for the study can be made available to others upon request and after an evaluation of confidentiality.

## References

[1] Rose JJ, Krishnan-Sarin S, Exil VJ, Hamburg NM, Fetterman JL, Ichinose F, Perez-Pinzon MA, Rezk-Hanna M, Williamson E (2023). Cardiopulmonary impact of electronic cigarettes and vaping products: a scientific statement from the American Heart Association. Circulation.

[2] Olfert IM, DeVallance E, Hoskinson H, Branyan KW, Clayton S, Pitzer CR, Sullivan DP, Breit MJ, Wu Z, Klinkhachorn P, Mandler WK, Erdreich BH, Ducatman BS, Bryner RW, Dasgupta P, Chantler PD (2018). Chronic exposure to electronic cigarettes results in impaired cardiovascular function in mice. Journal of Applied Physiology (1985).

[3] Kaplan A, Abidi E, Diab R, Ghali R, Al-Awassi H, Booz GW, Zouein FA (2022). Sex differences in cardiac remodeling post myocardial infarction with acute cigarette smoking. Biology of Sex Differences.

[4] Colivicchi F, Mocini D, Tubaro M, Aiello A, Clavario P, Santini M (2011). Effect of smoking relapse on outcome after acute coronary syndromes. American Journal of Cardiology.

[5] Noureddine S, Massouh A (2019). Factors associated with continued smoking in lebanese patients with acute coronary syndrome. Journal of Cardiovascular Nursing.

[6] Koçak A, Yıldırım O, Coşgun A, Türkkanı MH (2023). Factors affecting smoking cessation after acute myocardial infarction. Thoracic Research and Practice.

[7] Critchley JA, Capewell S (2003). Mortality risk reduction associated with smoking cessation in patients with coronary heart disease: A systematic review. JAMA.

[8] Adkins-Hempel M, Japuntich SJ, Chrastek M, Dunsiger S, Breault CE, Ayenew W, Everson-Rose SA, Nijjar PS, Bock BC, Wu WC, Miedema MD, Carlson BM, Busch AM (2023). Integrated smoking cessation and mood management following acute coronary syndrome: Protocol for the post-acute cardiac event smoking (PACES) trial. Addiction Science & Clinical Practice.

[9] Busch AM, Leavens EL, Wagener TL, Buckley ML, Tooley EM (2016). Prevalence, reasons for use, and risk perception of electronic cigarettes among post-acute coronary syndrome smokers. Journal of Cardiopulmonary Rehabilitation and Prevention.

[10] Wen X, Xia T, Li R, Qiu H, Yu B, Zhang Y, Wang S (2023). Trends in electronic cigarette use among us adults with a history of cardiovascular disease. JAMA Network Open.

[11] Martin TP, MacDonald EA, Elbassioni AAM, O'Toole D, Zaeri AAI, Nicklin SA, Gray GA, Loughrey CM (2022). Preclinical models of myocardial infarction: From mechanism to translation. British Journal of Pharmacology.

[12] Yang Y, Hao Y, Liu J, Yang N, Hu D, Sun Z, Zhao D, Liu J (2022). Practice of reperfusion in patients with ST-segment elevation myocardial infarction in China: Findings from the improving care for cardiovascular disease in china-acute coronary syndrome project. Chinese Medical Journal (England).

[13] Müller-Ehmsen J, Peterson KL, Kedes L, Whittaker P, Dow JS, Long TI, Laird PW, Kloner RA (2002). Rebuilding a damaged heart: Long-term survival of transplanted neonatal rat cardiomyocytes after myocardial infarction and effect on cardiac function. Circulation.

[14] Dai W, Shi J, Siddarth P, Zhao L, Carreno J, Kleinman MT, Herman DA, Arechavala RJ, Renusch S, Hasen I, Ting A, Kloner RA (2023). Effects of electronic cigarette exposure on myocardial infarction and no-reflow, and cardiac function in a rat model. Journal of Cardiovascular Pharmacology and Therapeutics.

[15] Osborn JW, Barber BJ, Quillen EW, Abram RJ, Cowley AW (1986). Chronic measurement of cardiac output in unanesthetized rats using miniature thermocouples. American Journal of Physiology.

[16] Brandli A (2015). Remote limb ischemic preconditioning: a neuroprotective technique in rodents. Journal of Visualized Experiments.

[17] Kenwright DA, Thomson AJ, Hadoke PW, Anderson T, Moran CM, Gray GA, Hoskins PR (2015). A protocol for improved measurement of arterial flow rate in preclinical ultrasound. Ultrasound International Open.

[18] Siddiqi TJ, Rashid AM, Siddiqi AK, Anwer A, Usman MS, Sakhi H, Bhatnagar A, Hamburg NM, Hirsch GA, Rodriguez CJ, Blaha MJ, DeFilippis AP, Benjamin EJ, Hall ME (2023). Association of electronic cigarette exposure on cardiovascular health: A Systematic review and meta-analysis. Current Problems in Cardiology.

[19] Mohammadi L, Han DD, Xu F, Huang A, Derakhshandeh R, Rao P, Whitlatch A, Cheng J, Keith RJ, Hamburg NM, Ganz P, Hellman J, Schick SF, Springer ML (2022). Chronic E-cigarette use impairs endothelial function on the physiological and cellular levels. Arteriosclerosis, Thrombosis, and Vascular Biology.

[20] Saavedra-Alvarez A, Pereyra KV, Toledo C, Iturriaga R, Del Rio R (2022). Vascular dysfunction in HFpEF: Potential role in the development, maintenance, and progression of the disease. Front Cardiovascular Medicine.

[21] Martinez-Morata I, Sanchez TR, Shimbo D, Navas-Acien A (2020). Electronic cigarette use and blood pressure endpoints: A systematic review. Current Hypertension Reports.

[22] Ward KD, Bliss RE, Vokonas PS, Garvey AJ (1993). Effects of smoking cessation on blood pressure. American Journal of Cardiology.

[23] Duarte DR, Minicucci MF, Azevedo PS, Matsubara BB, Matsubara LS, Novelli EL, Paiva SA, Zornoff LA (2009). The role of oxidative stress and lipid peroxidation in ventricular remodeling induced by tobacco smoke exposure after myocardial infarction. Clinics (São Paulo, Brazil).

[24] Chaumont M, de Becker B, Zaher W, Culié A, Deprez G, Mélot C, Reyé F, Van Antwerpen P, Delporte C, Debbas N, Boudjeltia KZ, van de Borne P (2018). Differential effects of E-cigarette on microvascular endothelial function, arterial stiffness and oxidative stress: A randomized crossover trial. Science and Reports.

[25] Skotsimara G, Antonopoulos AS, Oikonomou E, Siasos G, Ioakeimidis N, Tsalamandris S, Charalambous G, Galiatsatos N, Vlachopoulos C, Tousoulis D (2019). Cardiovascular effects of electronic cigarettes: A systematic review and meta-analysis. European Journal of Preventive Cardiology.

[26] Dai W, Kay GL, Kloner RA (2014). The therapeutic effect of cell transplantation versus noncellular biomaterial implantation on cardiac structure and function following myocardial infarction. Journal of Cardiovascular Pharmacology and Therapeutics.

[27] Dai W, Shi J, Gupta RC, Sabbah HN, Hale SL, Kloner RA (2014). Bendavia, a mitochondria-targeting peptide, improves postinfarction cardiac function, prevents adverse left ventricular remodeling, and restores mitochondria-related gene expression in rats. Journal of Cardiovascular Pharmacology.

[28] Ip M, Diamantakos E, Haptonstall K, Choroomi Y, Moheimani RS, Nguyen KH, Tran E, Gornbein J, Middlekauff HR (2020). Tobacco and electronic cigarettes adversely impact ECG indexes of ventricular repolarization: Implication for sudden death risk. American Journal of Physiology. Heart and Circulatory Physiology.

