# Supplementary material for: Effects of Electronic Cigarette Vaping on Cardiac and Vascular Function, and Post-myocardial Infarction Remodeling in Rats
Source: Cardiovasc Toxicol. 2024 Feb 10;24(2):199–208. doi: 10.1007/s12012-024-09835-8 (PMC10896768; doi:10.1007/s12012-024-09835-8)
Supplement: Supplementary file 1 — Supplementary file1 (DOCX 27 KB) [file 12012_2024_9835_MOESM1_ESM.docx]

**Supplementary material**

Exploratory analyses examining interaction of sex and exposure group on effects of vaping are presented in the Supplementary Material. Most major measures, such as cardiac function (Table 1), vascular function (Table 3), and LV infarct size and infarct expansion index (Table 5), were not differentially affected in males vs. females.

Supplementary Table 1: Cardiac function assessed by echocardiography in male and female rats, separately

| **Group** | **Air** | **Air** | **E-cig Nic+** | **E-cig Nic+** | **p value*** |
| --- | --- | --- | --- | --- | --- |
| **Sex** | **Male** | **Female** | **Male** | **Female** |  |
|  | n = 20 | n = 17 | n = 16 | n = 16 |  |
| LV Diastolic ID (mm) | 7.97 ± 0.23 | 7.71 ± 0.25 | 8.12 ± 0.26 | 7.93 ± 0.26 | 0.9 |
| LV Systolic ID (mm) | 5.73 ± 0.26 | 5.75 ± 0.29 | 5.68 ± 0.29 | 5.86 ± 0.29 | 0.8 |
| LVFS (%) | 28.4 ± 1.8 | 25.5 ± 1.9 | 31.5 ± 2.0 | 26.2 ± 2.0 | 0.5 |
| LV systolic wall thickness (mm) | 4.1 ± 0.2 | 3.3 ± 0.2 | 4.2 ± 0.2 | 3.6 ± 0.2 | 0.8 |
| LV diastolic wall thickness (mm) | 3.1 ± 0.2 | 2.7 ± 0.2 | 3.0 ± 0.1 | 2.7 ± 0.2 | 0.7 |

LV: left ventricular; ID: internal diameter; LVFS: left ventricular fractional shortening.

*Significance of the interaction term between exposure group and sex.

Supplementary Table 2: Hemodynamic parameters in male and female rats, separately

| **Group** | **Air** | **Air** | **E-cig Nic+** | **E-cig Nic+** | **p value*** |
| --- | --- | --- | --- | --- | --- |
| **Gender** | **Male** | **Female** | **Male** | **Female** |  |
|  | n = 20 | n = 17 | n = 16 | n = 16 |  |
| Systolic Pressure (mmHg) | 84 ± 2 | 90 ± 2 | 77 ± 3 | 85 ± 3 | 0.7 |
| Diastolic Pressure (mmHg) | 67 ± 2 | 70 ± 2 | 59 ± 2 | 65 ± 2 | 0.7 |
| Mean Pressure (mmHg) | 74 ± 2 | 80 ± 2 | 66 ± 2 | 74 ± 2 | 0.7 |
| Pulse Pressure (mmHg) | 17 ± 1 | 20 ± 1 | 18 ± 1 | 20 ± 1 | 0.8 |
| Heart Rate (BPM) | 258 ± 7 | 204 ± 7 | 244 ± 7 | 195 ± 7 | 0.8 |
| LV Pes (mmHg) | 82 ± 3 | 89 ± 3 | 89 ± 4 | 85 ± 4 | 0.1 |
| LV Ped (mmHg) | 4.4 ± 0.4 | 5.8 ± 0.4 | 3.7 ± 0.5 | 6.2 ± 0.5 | 0.2 |
| dP/dt max (mmHg/s) | 4178 ± 183 | 4761 ± 198 | 4780 ± 204 | 4604 ± 204 | 0.06 |
| dP/dt min (mmHg/s) | 3513 ± 192 | 3862 ± 208 | 4040 ± 215 | 3576 ± 215 | 0.06 |
| Tau (ms) | 15.1 ± 0.7 | 16.2 ± 0.7 | 14.2 ± 0.7 | 17.4 ± 0.7 | 0.1 |

BPM: beats per minute; LV Pes: left ventricular end-systolic pressure; LV Ped: left ventricular end-diastolic pressure.

*Significance of the interaction term between exposure group and sex.

Supplementary Table 3: Parameters of vascular function assessed by flow-mediated vasodilation of femoral artery in male and female rats, separately

| **Group** | **Air** | **Air** | **E-cig Nic+** | **E-cig Nic+** | **p value*** |
| --- | --- | --- | --- | --- | --- |
| **Gender** | **Male** | **Female** | **Male** | **Female** |  |
|  | n = 20 | n = 17 | n = 16 | n = 16 |  |
| Artery ID (mm) before occlusion | 0.64 ± 0.03 | 0.58 ± 0.03 | 0.58 ± 0.03 | 0.56 ± 0.03 | 0.4 |
| Flow velocity (CM/S) before occlusion | 55.9 ± 4.2 | 42.7 ± 4.4 | 54.2 ± 4.6 | 50.2 ± 4.6 | 0.3 |
| Flow rate (ml/min) before occlusion | 0.81 ± 0.07 | 0.35 ± 0.07 | 0.65 ± 0.08 | 0.28 ± 0.08 | 0.6 |
|  |  |  |  |  |  |
| Artery ID (mm) after reperfusion | 0.63 ± 0.02 | 0.57 ± 0.03 | 0.58 ± 0.03 | 0.50 ± 0.03 | 0.7 |
| Flow velocity (CM/S) after reperfusion | 64.0 ± 4.1 | 43.4 ± 4.6 | 56.0 ± 4.6 | 40.9 ± 4.6 | 0.5 |
| Peak flow rate (ml/min) after reperfusion | 2.54 ± 0.21 | 1.60 ± 0.23 | 1.93 ± 0.23 | 1.25 ± 0.23 | 0.6 |
|  |  |  |  |  |  |
| Δ of artery ID (%) | 36 ± 2% | 43 ± 3% | 42 ± 3% | 50 ± 3% | 0.8 |
| Δ of flow velocity (%) | 63 ± 4% | 42 ± 5% | 55 ± 5% | 40 ± 5% | 0.5 |
| Δ of flow rate (%) | 154 ± 21% | 60 ± 22% | 93 ± 23% | 25 ± 23% | 0.6 |

Δ = percentage of change from baseline to post-reperfusion

*Significance of the interaction term between exposure group and sex.

Supplementary Table 4: Postmortem parameters in male and female rats, separately

| **Group** | **Air** | **Air** | **E-cig Nic+** | **E-cig Nic+** | **p value*** |
| --- | --- | --- | --- | --- | --- |
| **Gender** | **Male** | **Female** | **Male** | **Female** |  |
|  | n = 20 | n = 13 | n = 16 | n = 12 |  |
| Postmortem LV volume (ml) | 0.864 ± 0.033 | 0.523 ± 0.042 | 0.810 ± 0.037 | 0.524 ± 0.043 | 0.5 |
| BW (grams) | 509 ± 8 | 270 ± 10 | 446 ± 9 | 265 ± 11 | 0.004 |
| Tibia length(mm) | 45 ± 0.3 | 40 ± 0.4 | 44 ± 0.3 | 39 ± 0.4 | 0.9 |
| Heart weight (grams) | 1.342 ± 0.036 | 0.797 ± 0.044 | 1.170 ± 0.040 | 0.801 ± 0.046 | 0.04 |
| Left ventricle weight (grams) | 1.115 ± 0.031 | 0.663 ± 0.039 | 0.969 ± 0.035 | 0.689 ± 0.041 | 0.02 |
| Right Ventricle Weight (grams) | 0.232 ± 0.008 | 0.131 ± 0.010 | 0.193 ± 0.009 | 0.120 ± 0.010 | 0.1 |
| Heart weight/BW | 0.0026 ± 0.0001 | 0.0030 ± 0.0001 | 0.0026 ± 0.0001 | 0.0030 ± 0.0001 | 0.5 |
| LV weight/BW | 0.0022 ± 0.0001 | 0.0025 ± 0.0001 | 0.0022 ± 0.0001 | 0.0026 ± 0.0001 | 0.2 |
| Heart weight/Tibia | 0.0298 ± 0.0008 | 0.0199 ± 0.0010 | 0.0267 ± 0.0009 | 0.0206 ± 0.0010 | 0.04 |
| LV weight/tibia | 0.0248 ± 0.0007 | 0.0166 ± 0.0009 | 0.0221 ± 0.0008 | 0.0177 ± 0.0009 | 0.03 |
| Lung wet weight (grams) | 1.497 ± 0.046 | 1.139 ± 0.057 | 1.514 ± 0.051 | 1.184 ± 0.059 | 0.8 |
| Lung dry weight (grams) | 0.237 ± 0.007 | 0.170 ± 0.008 | 0.227 ± 0.007 | 0.177 ± 0.009 | 0.3 |
| Lung wet/dry weight ratio | 6.351 ± 0.193 | 6.694 ± 0.240 | 6.745 ± 0.216 | 6.722 ± 0.250 | 0.4 |

LV: left ventricular; BW: Body weight.

*Significance of the interaction term between exposure group and sex.

Supplementary Table 5: Post-infarction LV remodeling parameters in male and female rats, separately

| **Group** | **Air** | **Air** | **E-cig Nic+** | **E-cig Nic+** | **p value*** |
| --- | --- | --- | --- | --- | --- |
| **Gender** | **Male** | **Female** | **Male** | **Female** |  |
|  | n = 20 | n = 13 | n = 16 | n =12 |  |
| Total LV area (mm^2^) | 111.8 ± 3.0 | 78.2 ± 3.7 | 102.4 ± 3.3 | 81.8 ± 3.8 | 0.1 |
| LV cavity area (mm^2^) | 58.1 ± 2.1 | 34.4 ± 2.6 | 50.4 ± 2.4 | 37.2 ± 2.7 | 0.04 |
| Infarct size (% of LV circumference) | 42.7 ± 2.2 | 41.8 ± 2.7 | 39.2 ± 2.5 | 41.5 ± 2.9 | 0.5 |
| LV infarcted wall thickness (mm) | 0.8 ± 0.05 | 0.75 ± 0.07 | 0.85 ± 0.06 | 0.67 ± 0.07 | 0.3 |
| LV non-infarcted wall thickness (mm) | 2.0 ± 0.07 | 1.94 ± 0.09 | 1.98 ± 0.08 | 2.08 ± 0.09 | 0.4 |
| Infarct expansion index | 1.38 ± 0.10 | 1.22 ± 0.13 | 1.29 ± 0.11 | 1.55 ± 0.3 | 0.1 |

LV: left ventricular.

*Significance of the interaction term between exposure group and sex.
